# Supplementary figures and images for: Placozoan secretory cell types implicated in feeding, innate immunity and regulation of behavior
Source: PLoS One. 2025 Apr 22;20(4):e0311271. doi: 10.1371/journal.pone.0311271 (PMC12013895; doi:10.1371/journal.pone.0311271)

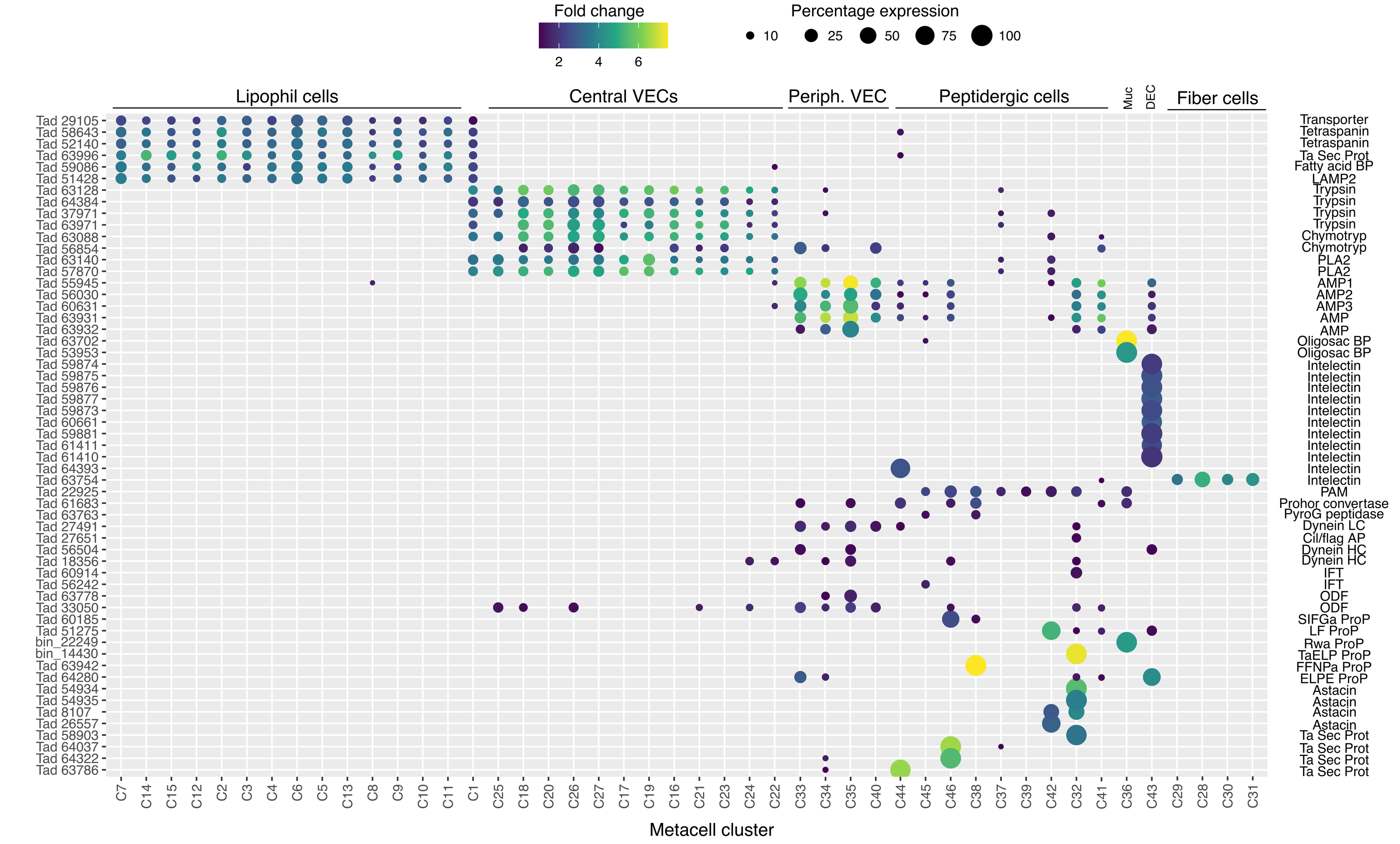

Supplement: S1 Fig — Data are from [30]. Cell types are identified based on data from the present study and [6,18,24,30]. Dot color represents fold change of gene expression and dot size represents percentage of the total UMI in the given metacell. (TIF) [file pone.0311271.s001.tif]

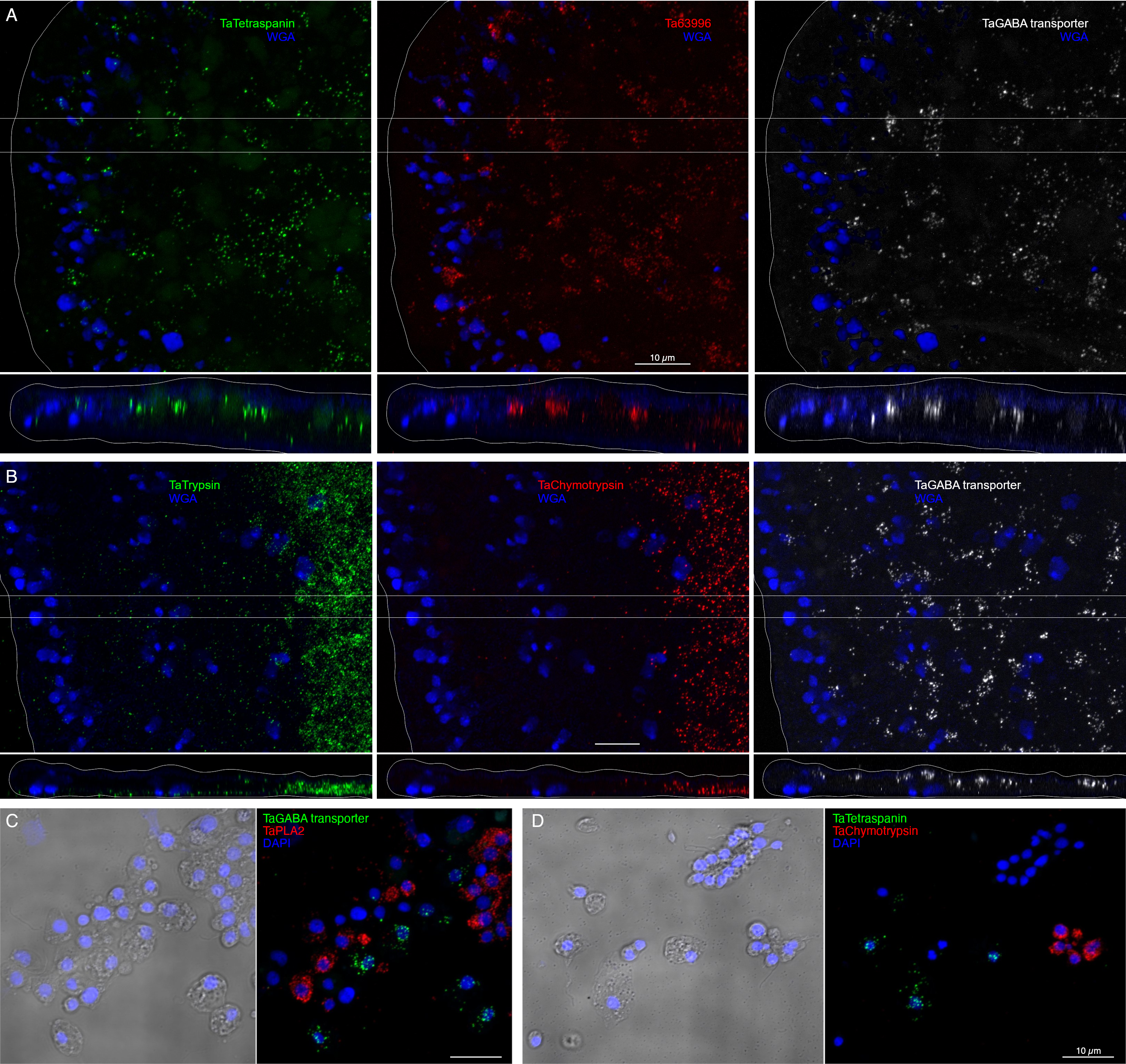

Supplement: S2 Fig — . (A, B) Separated channels corresponding to horizontal (xy) and vertical (xz) projections of color-merged FISH images of TH1 wholemounts in Fig 2A and B. (C, D) Separated DIC and fluorescence channels of the merged images of dissociated cells in Fig 2C and D. (TIF) [file pone.0311271.s002.tif]

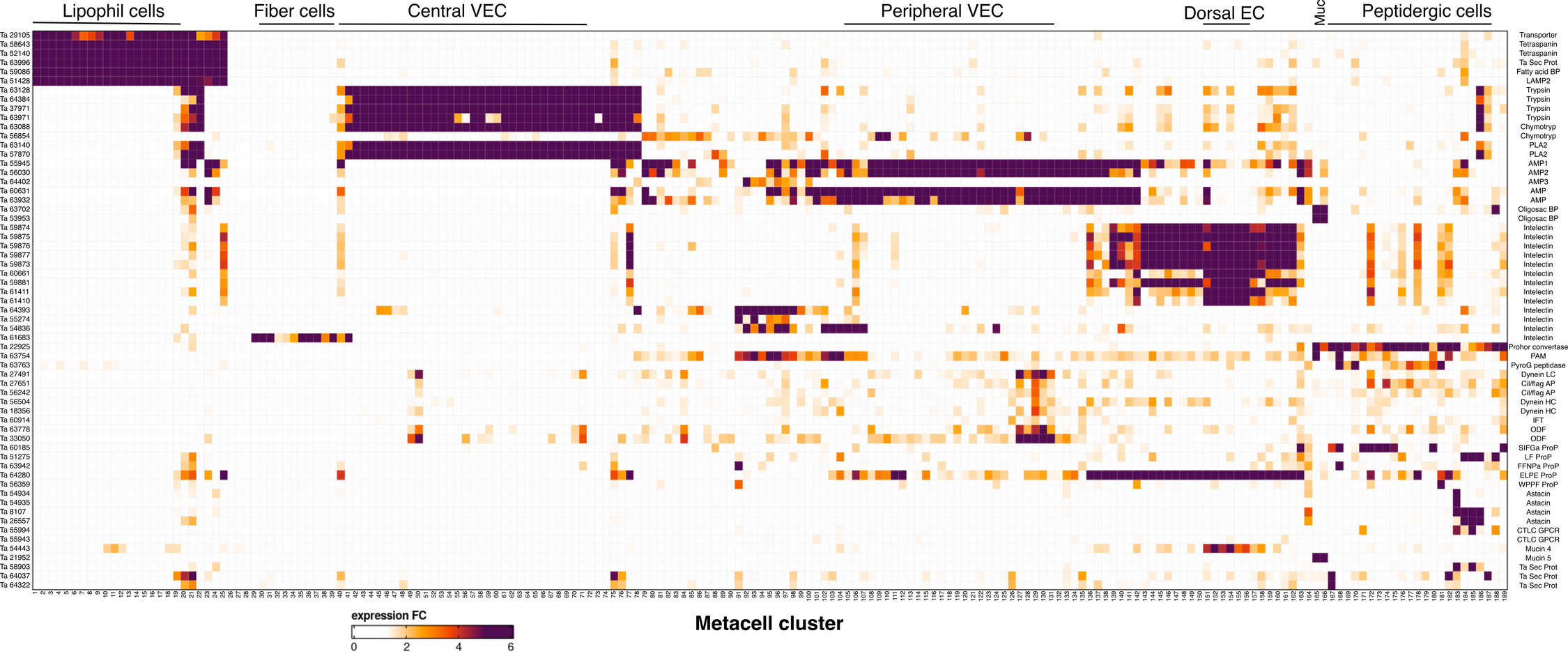

Supplement: S3 Fig — Cell types are identified based on data from the present study and [6,18,30]. Expression data from: https://sebelab.crg.eu/placozoa_cell_atlas/. (TIF) [file pone.0311271.s003.tif]

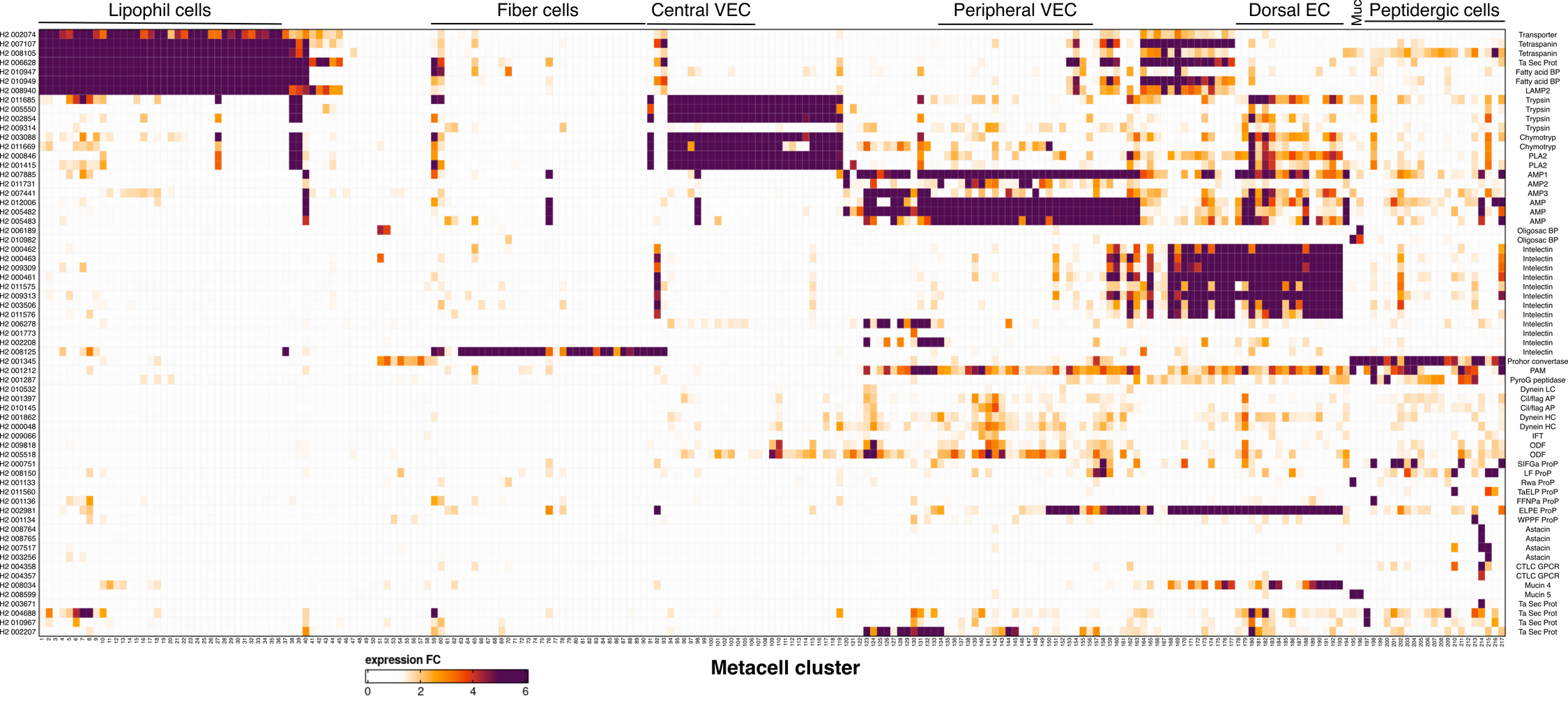

Supplement: S4 Fig — Expression data from: https://sebelab.crg.eu/placozoa_cell_atlas/. (TIF) [file pone.0311271.s004.tif]

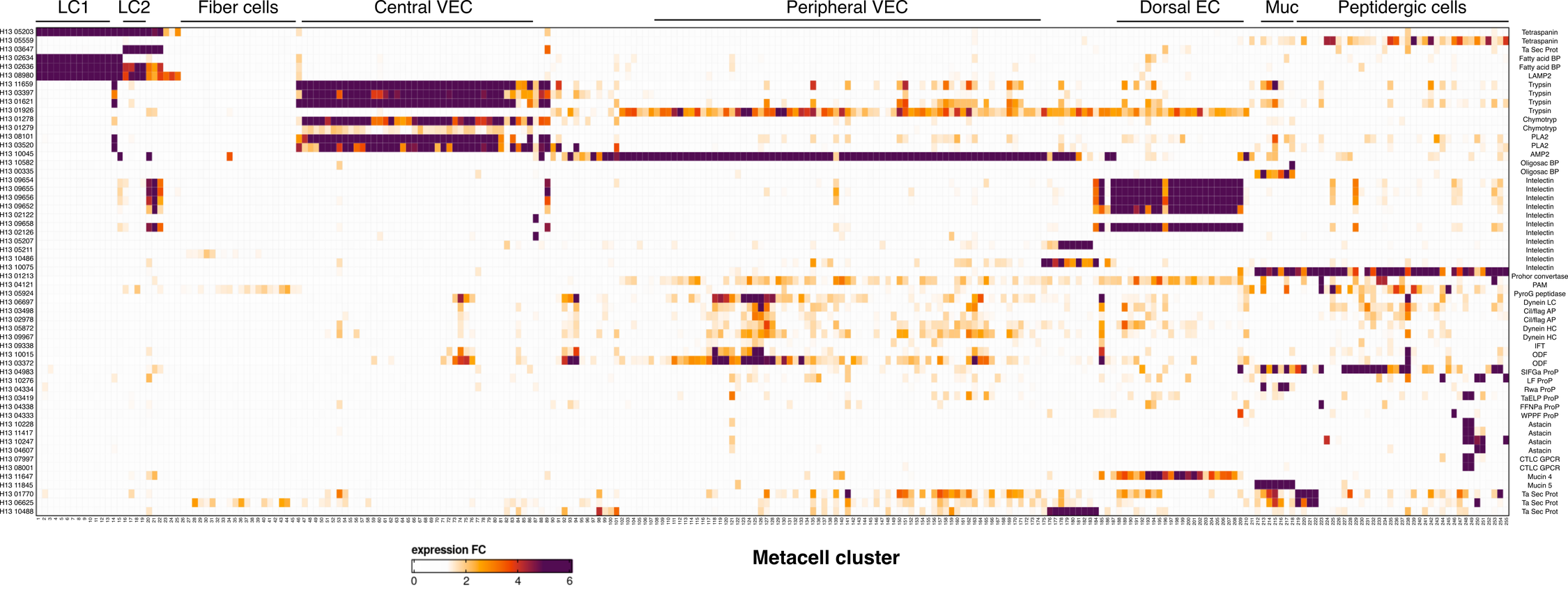

Supplement: S5 Fig — Expression data from: https://sebelab.crg.eu/placozoa_cell_atlas/. (TIF) [file pone.0311271.s005.tif]

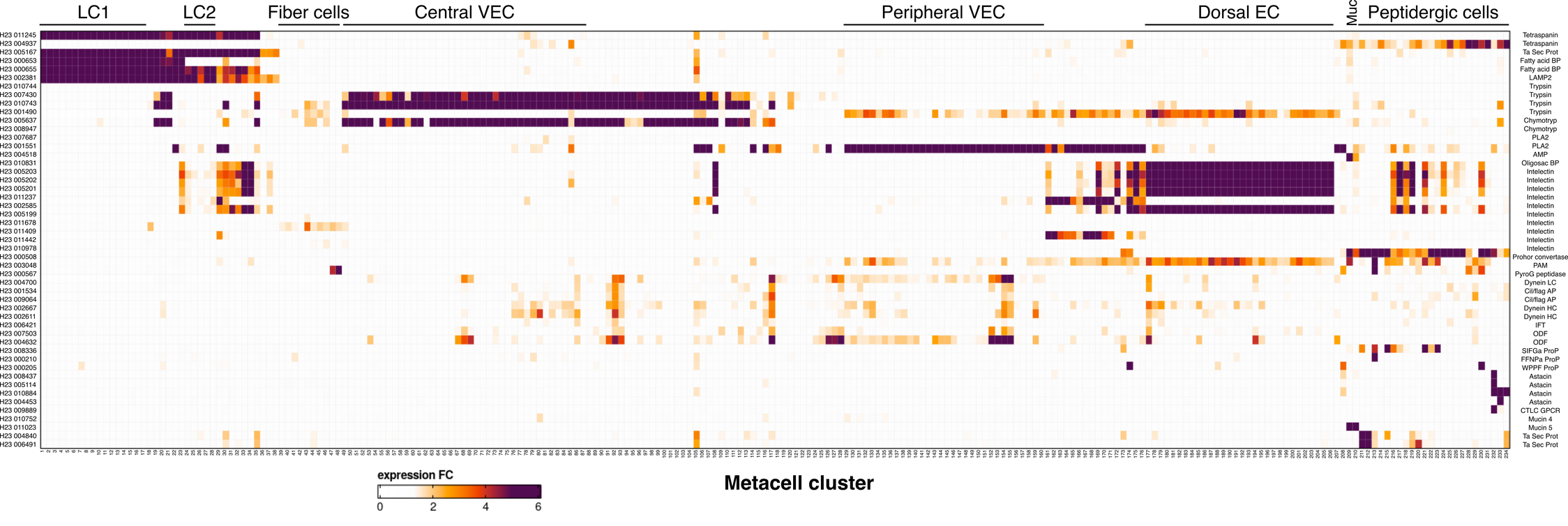

Supplement: S6 Fig — Expression data from: https://sebelab.crg.eu/placozoa_cell_atlas/. (TIF) [file pone.0311271.s006.tif]

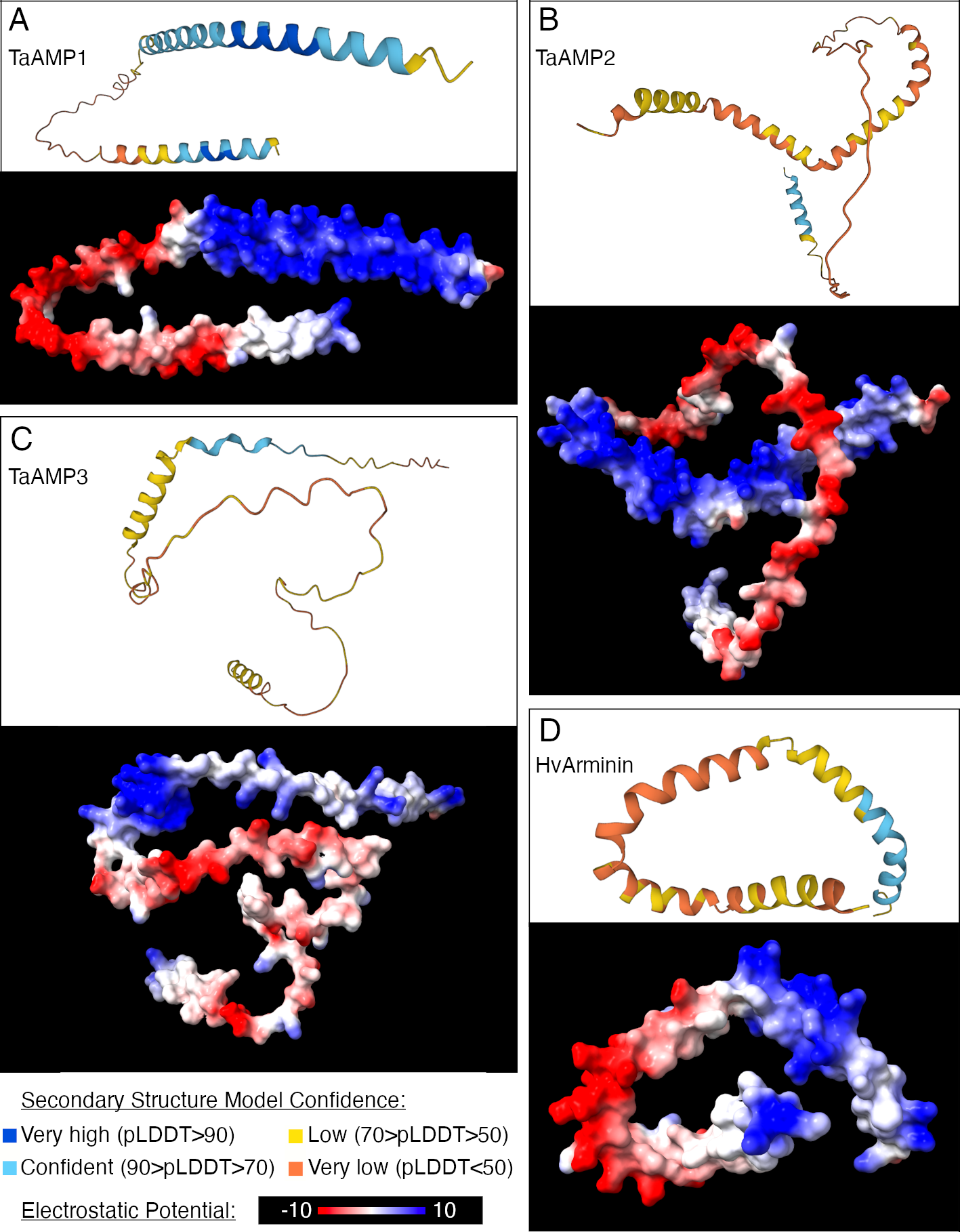

Supplement: S7 Fig — Secondary structures are predicted with Alpha Fold and peptide surfaces are colored by electrostatic potential with ChimeraX. (TIF) [file pone.0311271.s007.tif]

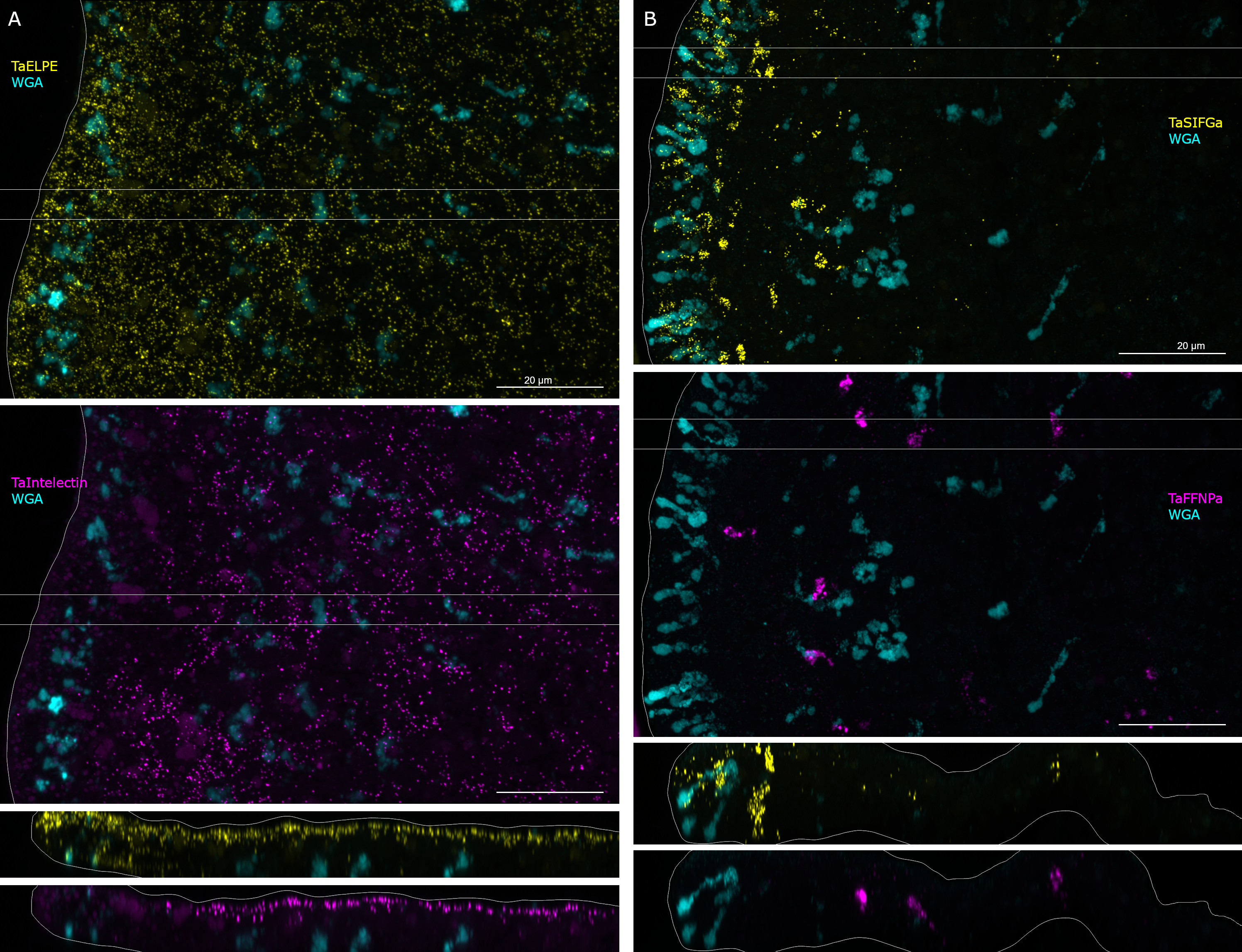

Supplement: S9 Fig — (TIF) [file pone.0311271.s009.tif]

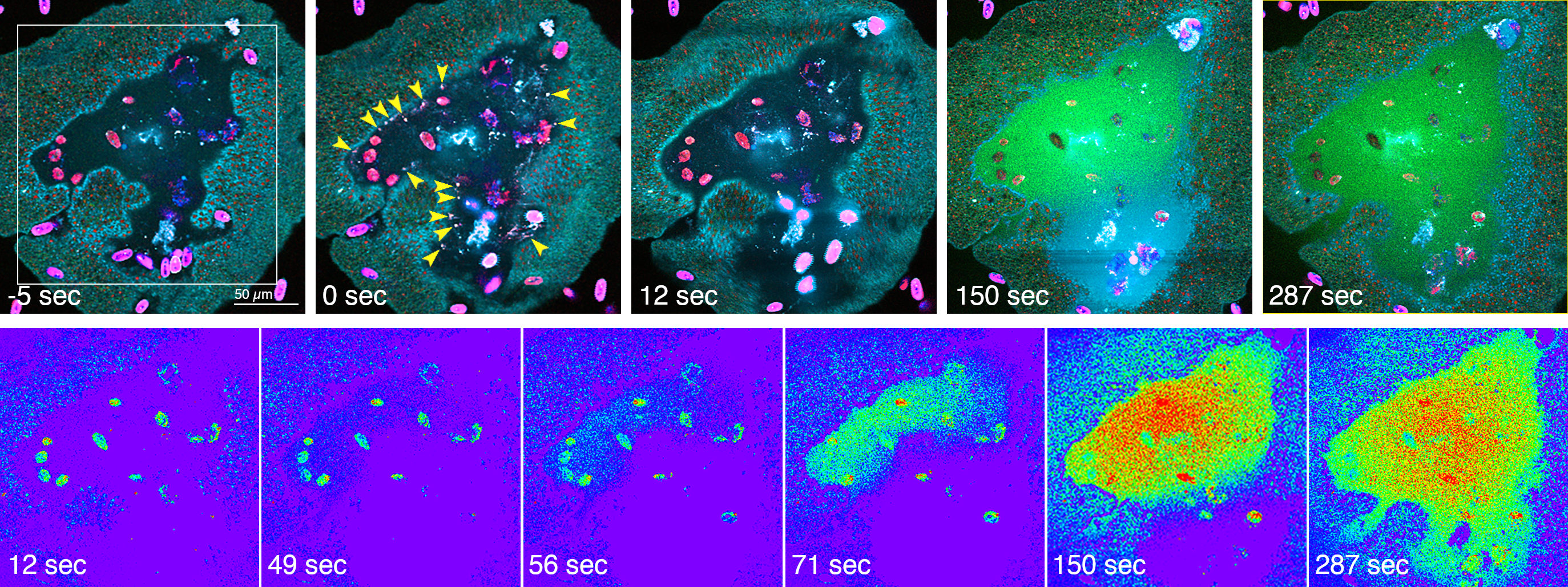

Supplement: S10 Fig — Lipophil cell granules were labeled with LipidTOX (red). The fluorescent membrane dye FM1–43 (cyan) was added to the seawater to label cell membranes and the contents of LC granules. BZiPAR, a fluorescent indicator of trypsin activity (green), was added to detect secretion of trypsin. Algae are visible by autofluorescence and FM1–43 staining (pink in merged images). At the beginning of the sequence, the peripheral part of the animal was closely attached to the substrate, while the central part was invaginated, forming a feeding pocket enclosing algae. At t=0 sec, lipophil granule secretion was evident due to the appearance of small FM1–43-stained spots (cyan/white; arrowheads) in the feeding pocket. At t=12 sec, several algae (pink) in the feeding pocket were lysed and released material stained by FM1–43 (cyan). By 150–287 sec, trypsin activity was evident in the feeding pocket and the lysed algae were decomposing. Bottom panels show intensity-coded images of BZiPAR trypsin activity indicator at sequential timepoints for the boxed region. (TIF) [file pone.0311271.s010.tif]
