# Supplementary material for: Placozoan secretory cell types implicated in feeding, innate immunity and regulation of behavior
Source: PLoS One. 2025 Apr 22;20(4):e0311271. doi: 10.1371/journal.pone.0311271 (PMC12013895; doi:10.1371/journal.pone.0311271)
Supplement: Attachment — Submitted filename: Response to Reviewers 2.docx [file pone.0311271.s015.docx]

**Manuscript PONE-D-24-38008	January 16, 2025**

Dear Dr. Schubert,

Thank you for giving us the opportunity to submit a second revision of the manuscript “Placozoan secretory cell types implicated in feeding, innate immunity and regulation of behavior” for publication in the PLOS One. We appreciate the time and effort that you and the reviewers dedicated to providing feedback on our manuscript and are grateful for resulting improvements to our paper. We modified the Introduction and Discussion to make them more concise and made several additional changes for clarity. Those changes are highlighted within “Revised Manuscript with Track Changes” and explained in “Response to Reviewers” (red font).

The reference list in “Revised Manuscript with Track Changes” includes references that were deleted so the numbering is incorrect. The reference list and numbering in “Revised Manuscript” is correct.

Thank you for your attention,

Carolyn Smith

NINDS/NIH

36 Convent Drive,

Bethesda, MD 20892

**Reviewers' Comments to the Authors:**

Reviewer #1: The addition of separated channel images as supplementary figures has indeed improved the interpretation of the multiplex FISH data.
Additional images, such as Fig1, do make the manuscript more appealing to a wider audience of non Placozoan specialists (although Fig1C should be more appropriately described as 'A drawing of main cell types in TH1 as observed by Transmitted Electron Microscopy').

We modified legend for Fig1C as suggested.

The gene nomenclature has been harmonized throughout the manuscript, making it much easier to read.
Mistakes and typos have been mostly corrected (although 'Rhodamonas' should be corrected to 'Rhodomonas').

Corrected.

The authors chose to keep lots of data and discussion which in my opinion are not strictly pertinent or excessively speculative. This is a legitimate choice but unfortunately it distracts the readers' attention away from the most interesting and remarkable achievement of this work, i.e. the generation of a detailed, single-cell level atlas of gene expression patterns in a little-studied and not easily amenable metazoan taxon. Personally, I still believe that a more concise and matter-of-fact version of the manuscript would be preferable and have a much stronger and wider impact. However, I agree that my should be balanced with that of reviewer n°2 and with the editor's advice.

We modified the Introduction and Discussion for clarity and to make them more concise.

Line numbers refer to “Revised Manuscript with Track Changes”. Note that the reference numbering in this document is incorrect because the reference list includes deleted references.

**Introduction**

Modified text at lines 79-80, 138, 159-162.

Deleted text at lines 101, 105, 116, 131-133, 137-139, 144, 162.

**Discussion**

Modified text at lines 747-749, 830-835, 886, 905, 944-945, 984, 1014-1015, 1113-1122.

Deleted text at lines 764, 806, 835, 850, 881, 883, 946, 984, 986, 1014, 1017, 1122.

Conclusions section of the Discussion was modified for clarity and to better explain the relevance of our work to efforts to understand the evolution of secretory cells (lines 1125-1176).

Reviewer #2: I think the authors greatly improved my main criticism, which was that they needed to do a better job connecting with a broader audience. They improved that with the addition of Figure 1 and by creating the summary figures as I recommended. My only point that was not addressed was comparing existing expression pattern data (like Trox-2) or other markers to help explain the domains of patterning, but I understand if this extends away from the focus of the paper. Overall I think this work would be a nice fit for PLOS One.
